# Supplementary material for: 4H silicon carbide bulk acoustic wave gyroscope with ultra-high Q-factor for on-chip inertial navigation
Source: Commun Eng. 2024 Jun 25;3:87. doi: 10.1038/s44172-024-00234-z (PMC11199606; doi:10.1038/s44172-024-00234-z)
Supplement: Supplementary file 2 — Supplementary Information [file 44172_2024_234_MOESM2_ESM.pdf]

4H Silicon Carbide Bulk Acoustic Wave Gyroscope with Ultra-High Q-factor for On-Chip Inertial Navigation – supplementary information

Zhenming Liu[1], Yaoyao Long[1], Charlotte Wehner[1], Haoran Wen[2], and Farrokh Ayazi[1]

[1] School of Electrical and Computer Engineering, Georgia Institute of Technology, Atlanta, GA 30308, USA

[2] StethX Microsystems Inc., Atlanta, GA, 30308, USA

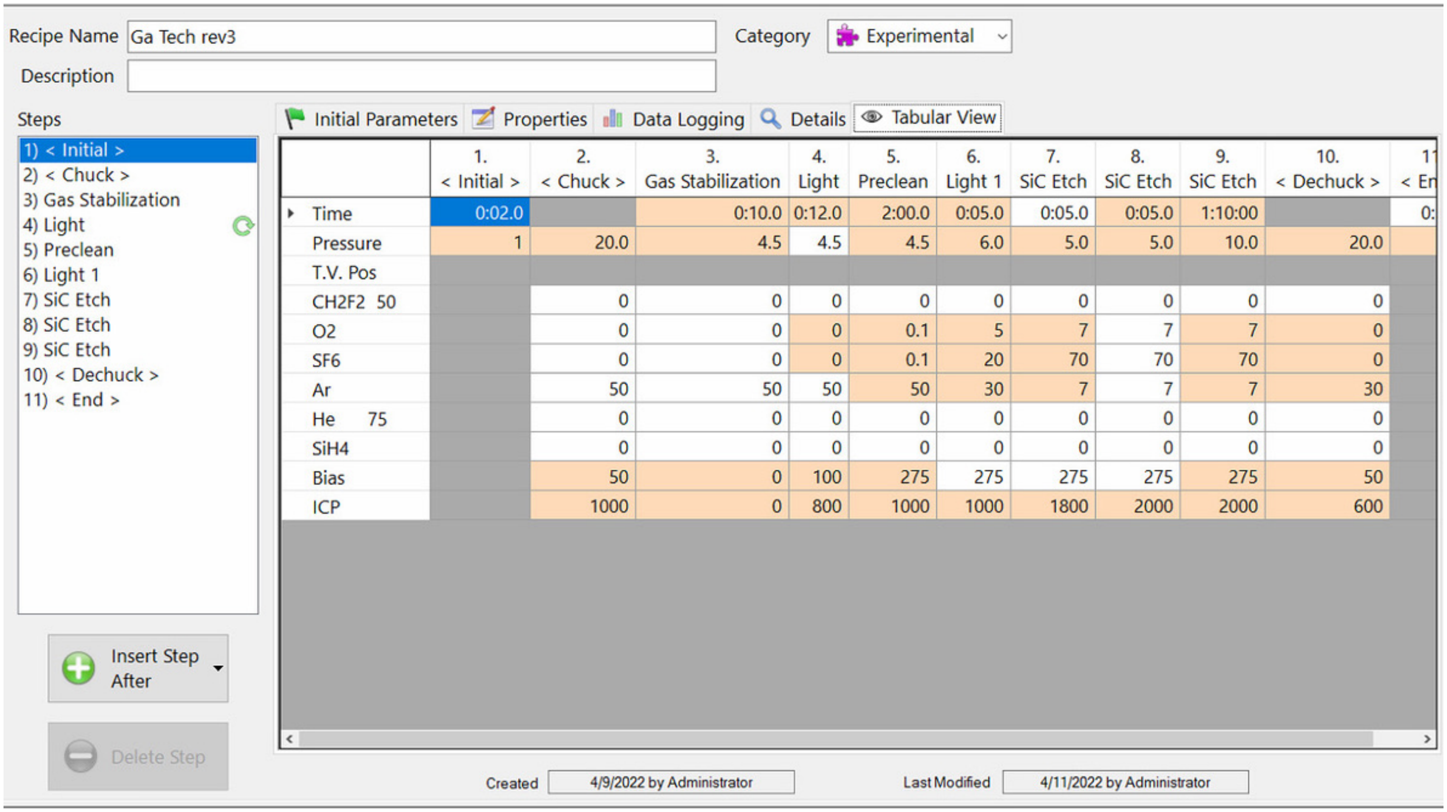

	1. < Initial >	2. < Chuck >	3. Gas Stabilization	4. Light	5. Preclean	6. Light 1	7. SiC Etch	8. SiC Etch	9. SiC Etch	10. < Dechuck >	11 < En
Time	0:02.0		0:10.0	0:12.0	2:00.0	0:05.0	0:05.0	0:05.0	1:10:00		0:
Pressure	1	20.0	4.5	4.5	4.5	6.0	5.0	5.0	10.0	20.0	
T.V. Pos											
CH2F2 50		0	0	0	0	0	0	0	0	0	
O2		0	0	0	0.1	5	7	7	7	0	
SF6		0	0	0	0.1	20	70	70	70	0	
Ar		50	50	50	50	30	7	7	7	30	
He 75		0	0	0	0	0	0	0	0	0	
SiH4		0	0	0	0	0	0	0	0	0	
Bias		50	0	100	275	275	275	275	275	50	
ICP		1000	0	800	1000	1000	1800	2000	2000	600	

Supplementary Fig. 1| SiC DRIE recipe.

Supplementary Note 1:

The key to achieving a pristine etching profile is to control the nickel fluoride passivation by-product from the SF6 and nickel mask reaction. Such passivation profile is mostly dependent on electrode temperature and bias power. A lower temperature and higher bias power typically increase the passivation growth rate, which is helpful in preserving the sidewall smoothness but could also pinch the trench as passivation closes the gap; on the contrary, if the passivation growth rate is too slow, significant damage can be seen on the sidewall as it is directly exposed to the plasma.

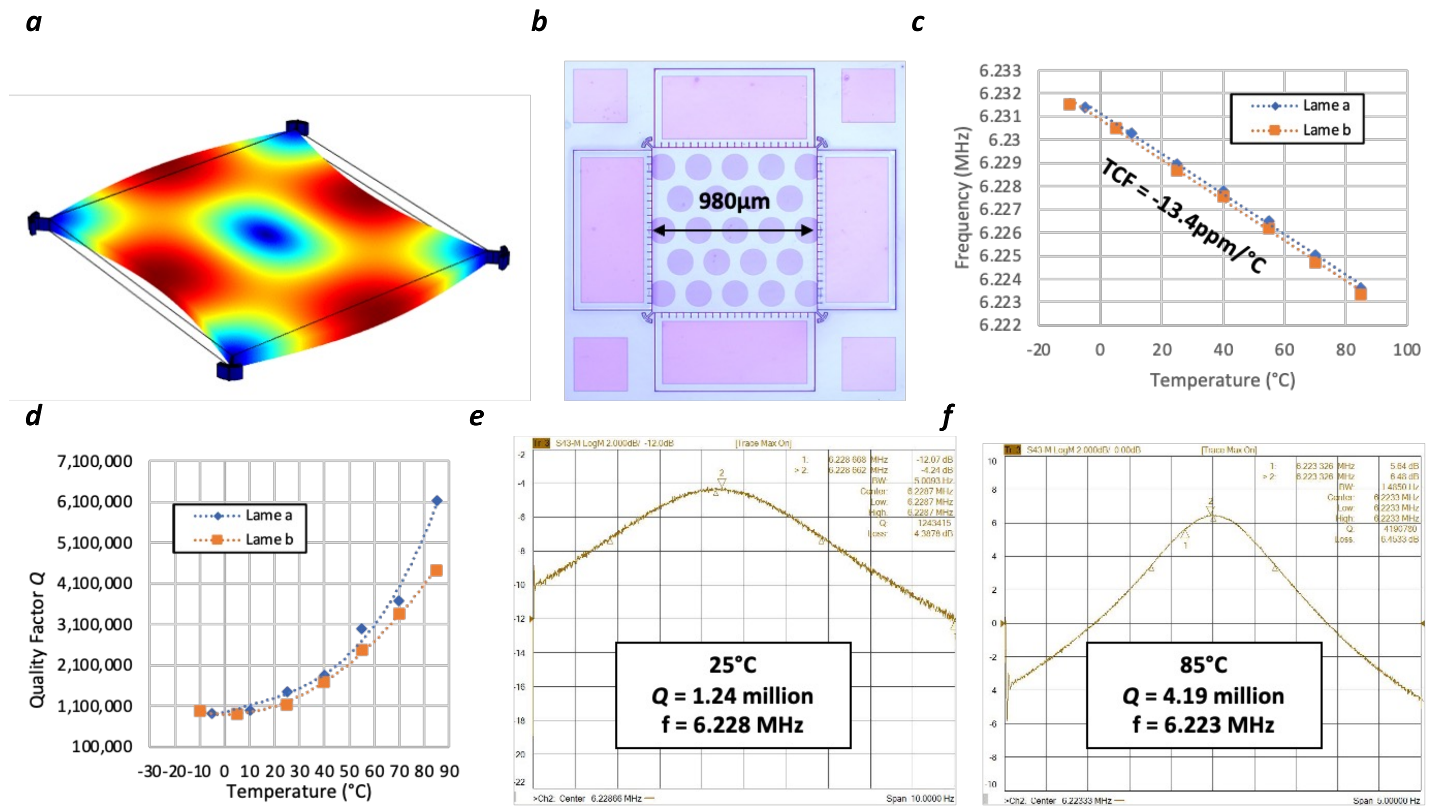

Supplementary Fig. 2| 4H-SiC Lame resonator. a, the mode shape **b,** the optical picture of the fabricated lame resonator **c,** a linear TCF of -13.4ppm/°C was extracted from two separated 4H-SiC lame devices. **d,** a positive nonlinear TCQ was observed in 4H-SiC Lame resonators, similar to the disk resonant gyroscopes. **e**, at room temperature, Q of 1.24 million was measured. **f**, at 85°C, Q of the same device increased to 4.19 million.

Supplementary Note 2:

The lame resonators are fabricated on the same SiC-on-insulator wafers as the BAW disk resonant gyroscopes from the main text, it can bring more insight of the 4H-SiC as an acoustic material for MEMS devices. This resonator is designed with side supported decoupling network located in the four nodes, as shown in supplementary Fig.1a, with anchor Q in the order of 10^{11} based on FEA simulation. Fig.1b shows the fabricated devices. The linear TCF shown in Fig.1c, with values close to that of the center-supported disk resonators, proves the resonator is stress-free. The measured Q increases with temperature, mainly limited by the TED due to the sidewall roughness, shown in Fig.1d. Such an increase in Q is very similar to the disk resonators. Fig.1e&f show the frequency response measurement at room temperature and 85°C, a significant Q increase was observed from 1.24 million to 4.19 million.

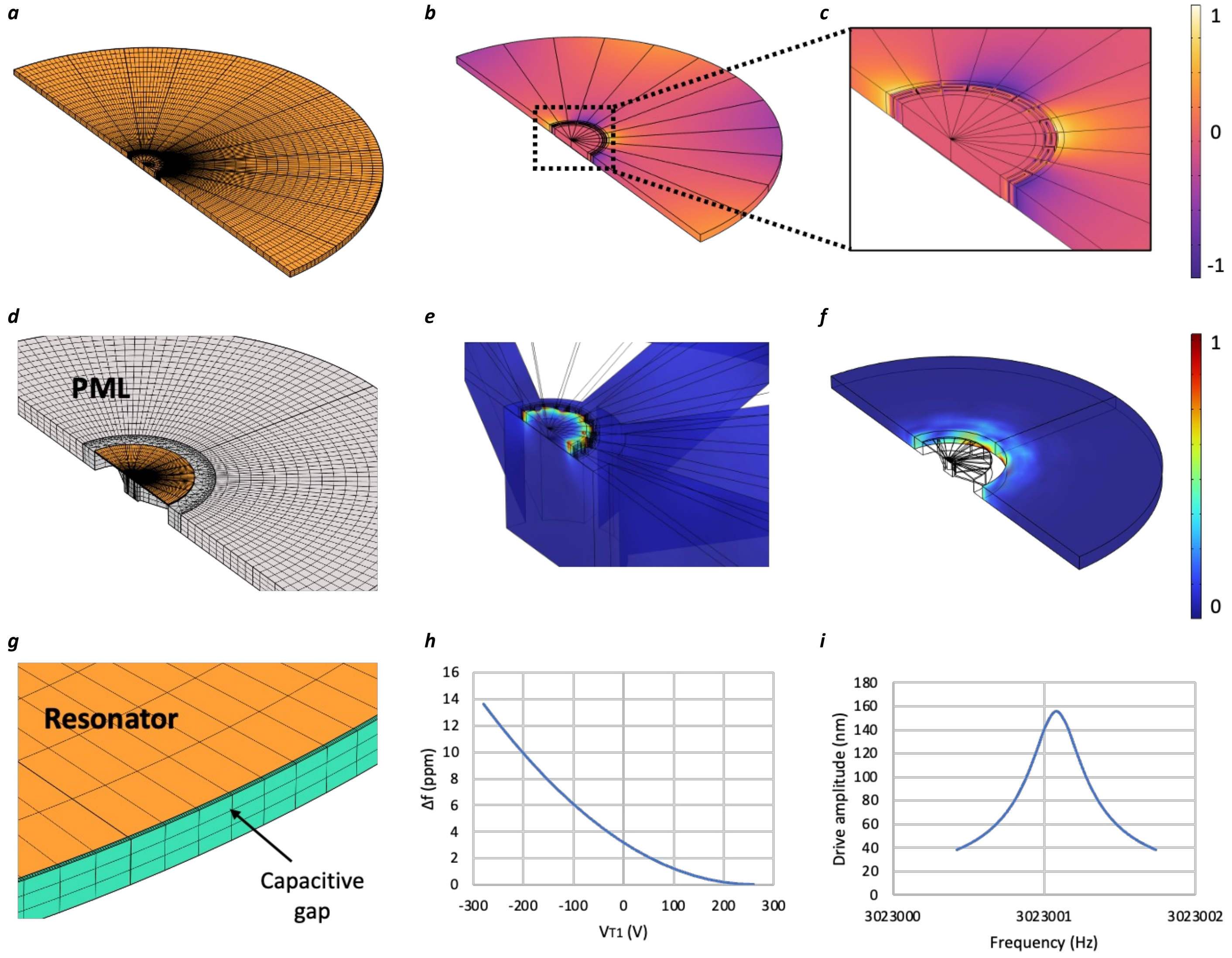

Supplementary Fig. 3| Finite element analysis simulation. a, the reconstruction of the 4H-SiC disk resonator and its meshing condition using COMSOL Multiphysics® software **b,** the normalized temperature perturbation result in the TED simulation **c,** the zoom-in view at the decoupling network where the temperature perturbation is maximum. **d,** reconstruction of the 4H-SiC disk resonator anchored on a silicon handle layer, surrounded by a perfect match layer (PML), and its meshing condition. **e**, the exaggerated displacement at the anchor location. **f,** the acoustic wave propagation in the PML layer. **g,** reconstruction of the capacitive gap and the meshing. **h,** tuning range analysis. Vp of 260V is applied to the disk resonator, and V_{T1} is applied to a total of 4 tuning electrodes. A total of 460V voltage difference is needed to mode match a device with a 10 ppm initial frequency split. **i,** the driving amplitude analysis. For a 3mm 4H-SiC BAW disk resonator with 4.6 million *Q*, 3.5μm wide transduction gap, and 260V DC bias voltage, using 650mV AC voltage can achieve 159nm driving amplitude.

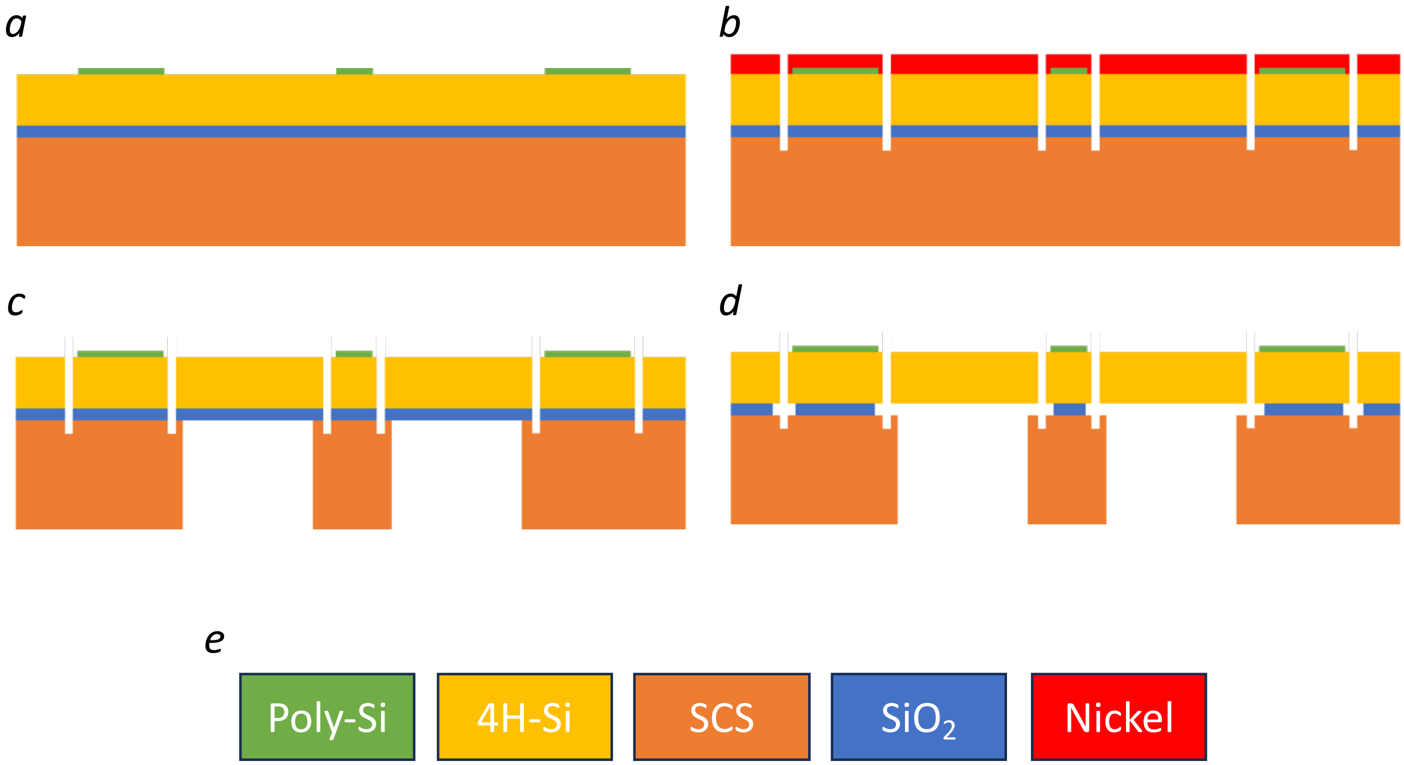

Supplementary Fig. 4| Device process flow on the SiC-on-insulator platform. a, deposit and pattern a layer of doped polycrystalline silicon thin film (500nm~1μm) on SiC **b,** pattern nickel mask (~7μm) and etch through the SiC layer (40μm) to define the resonator geometry and transduction gaps **c,** etch the release hole on the backside silicon handle layer (500μm). **d,** dice the wafer into individual dies, anneal to strengthen the bottom oxide bond, and RTP to active the ohmic contact between the poly-Si pad and SiC device. Release the device in hydrofluoric acid to remove the bottom silicon oxide layer (4μm) **e,** color code for the process flow.

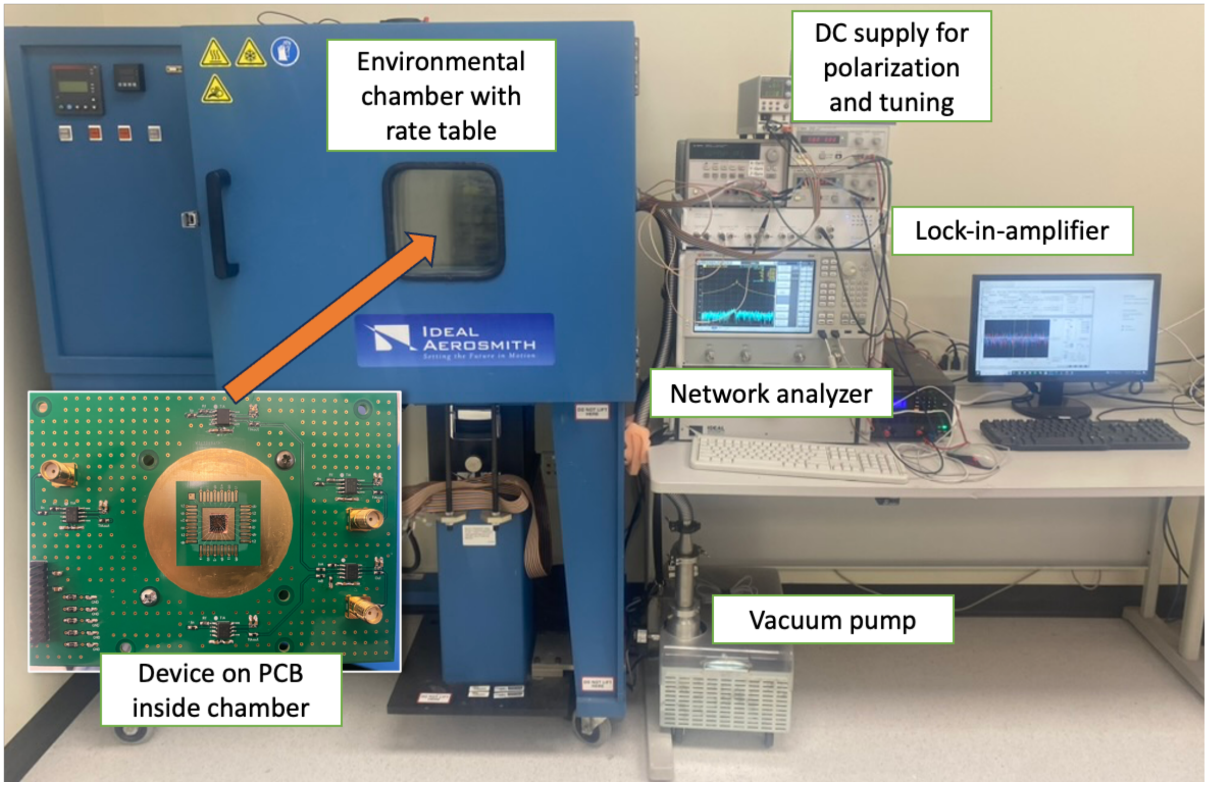

Supplementary Fig. 5| Device testing setup.

Supplementary Note 3:

The disk resonant gyroscope in the main text has an initial frequency split of 9.3 ppm and cross-mode isolation of 16 dB. It is mode matched using the following condition: $V_p = V_{T2} = V_{Qb} = 260V$; $V_{T1} = -172V$; $V_{Qa} = 82.5V$. During gyroscope measurement, V_{Qa} is cascaded to the DC output from the lock-in-amplifier for close loop quadrature cancelation.

Supplementary Table 1 | Literature review of planer silicon MEMS gyroscope since 1988

Year	ARW	Institute	Reference
1988	N.A.	Draper, USA	1
1993	83	Draper, USA	2
1995	120	Murata, Japan	3
1997	2	UC Berkey, USA	4
1997	6.3	JPL & UCLA, USA	5
1998	0.27	HSG-IMIT, German	6
1998	0.05	U Mich, USA	7
1999	0.14	HSG-IMIT, German	8
2002	0.1	JPL, USA	9
2003	0.13	BAE system, USA	10
2004	1	Morgan Research Corp, USA	11
2004	0.6	Seoul National University, Korea	12
2004	0.072	Seoul National University, Korea	13
2005	0.3	Chemnitz University of Technology	14
2006	0.14	Cairo University, Egypt	15
2007	0.045	Ga Tech, USA	16
2008	0.03	Ga Tech, USA	17
2008	0.115	Middle East Technical University	18
2008	0.045	Ga Tech	19
2009	0.09	Ga Tech	20
2011	0.06	UC Irvine	21
2011	0.06	U Mich	22
2011	0.05	National University of Defense Technology	23
2012	0.09	U Mich	24
2012	0.014	Middle East Technical University	25
2012	0.06	UC Irvine	26
2013	0.36	UC Davis	27
2013	0.07	UC Irvine	28
2014	0.034	Stanford University	29
2014	0.08	UC Irvine	30
2015	0.14	South East University	31
2015	0.067	Nanjing University of Science and Technology	32
2015	0.1	Ajou University	33
2016	0.015	UC Irvine	34
2016	0.045	UC Irvine	35
2016	0.39	Ga Tech	36
2017	0.63	Ga Tech	37
2017	0.25	Analog Device	38
2017	0.15	CMU	39
2018	0.01	National University of Defense Technology	40
2018	0.2	GLOBALFOUNDRIES	41
2018	0.135	Newcastle University	42
2019	0.776	North University of China	43
2019	0.15	Northrop Grumman LITEF GmbH	44
2019	0.038	National University of Defense Technology	45
2020	0.06	Ga Tech	46
2020	0.86	Ga Tech	47
2020	0.032	UC Irvine	48
2021	0.02	Peking University	49

2021	0.923	Chinese Academy of Sciences	50
2021	0.05	Northwestern Polytechnical University	51
2022	0.145	Ga Tech	52
2022	0.027	University of Cambridge	53
2022	0.017	Panasonic	54
**2023**	**0.005**	**This work**	
**2024**	**0.0008**	**This work w/ low noise ASIC**	

Supplementary Reference:

[1] Boxenhorn, Burton, and Paul Greiff. "A vibratory micromechanical gyroscope." Guidance, Navigation and Control Conference. 1988.

[2] Bernstein, Jon, et al. "A micromachined comb-drive tuning fork rate gyroscope." [1993] Proceedings IEEE Micro Electro Mechanical Systems. IEEE, 1993.

[3] Tanaka, K., et al. "A micromachined vibrating gyroscope." Sensors and Actuators A: Physical 50.1-2 (1995): 111-115.

[4] Juneau, Thor, A. P. Pisano, and James H. Smith. "Dual axis operation of a micromachined rate gyroscope." Proceedings of International Solid State Sensors and Actuators Conference (Transducers' 97). Vol. 2. IEEE, 1997.

[5] Tang, Tony K., et al. "A packaged silicon MEMS vibratory gyroscope for microspacecraft." Proceedings IEEE The Tenth Annual International Workshop on Micro Electro Mechanical Systems. An Investigation of Micro Structures, Sensors, Actuators, Machines and Robots. IEEE, 1997.

[6] Geiger, W., et al. "A new silicon rate gyroscope." Sensors and Actuators A: Physical 73.1-2 (1999): 45-51.

[7] Ayazi, Farrokh, and Khalil Najafi. "Design and fabrication of high-performance polysilicon vibrating ring gyroscope." Proceedings MEMS 98. IEEE. Eleventh Annual International Workshop on Micro Electro Mechanical Systems. An Investigation of Micro Structures, Sensors, Actuators, Machines and Systems (Cat. No. 98CH36176. IEEE, 1998.

[8] Geiger, W., et al. "A new silicon rate gyroscope." Sensors and Actuators A: Physical 73.1-2 (1999): 45-51.

[9] Bae, S., et al. "JPL's MEMS gyroscope fabrication, 8-electrode tuning and performance results." Digest, Solid-State Sensors and Actuators Workshop. 2002.

[10] Fountain, J. Richard. "Characteristics and Overview of a Silicon Vibrating Structure Gyroscope." Advances in Navigation Sensors and Integration Technology (2003).

[11] Kranz, Michael, et al. "Performance of a silicon-on-insulator MEMS gyroscope with digital force feedback." PLANS 2004. Position Location and Navigation Symposium (IEEE Cat. No. 04CH37556). IEEE, 2004.

[12] Kim, Jongpal, et al. "A planar, x-axis, single-crystalline silicon gyroscope fabricated using the extended SBM process." 17th IEEE International Conference on Micro Electro Mechanical Systems. Maastricht MEMS 2004 Technical Digest. IEEE, 2004.

[13] Kim, Jongpal, et al. "Robust SOI process without footing and its application to ultra high-performance microgyroscopes." Sensors and Actuators A: Physical 114.2-3 (2004): 236-243.

[14] Hiller, K., et al. "Novel high precision micromachined gyroscope."

[15] Sharaf, Abdelhameed, Sherif Sedky, and SE-D. Habib. "Complete analysis of a novel fully symmetric decoupled micromachined gyroscope." 2006 International Conference on MEMS, NANO, and Smart Systems. IEEE, 2006.

[16] Sharma, Ajit, Mohammad Faisal Zaman, and Farrokh Ayazi. "A 104-dB dynamic range transimpedance-based CMOS ASIC for tuning fork microgyroscopes." IEEE Journal of Solid-State Circuits 42.8 (2007): 1790-1802.

[17] Sharma, Ajit, et al. "A 0.1°/HR bias drift electronically matched tuning fork microgyroscope." 2008 IEEE 21st International Conference on Micro Electro Mechanical Systems. IEEE, 2008.

[18] Alper, Said Emre, Yuksel Temiz, and Tayfun Akin. "A compact angular rate sensor system using a fully decoupled silicon-on-glass MEMS gyroscope." Journal of Microelectromechanical systems 17.6 (2008): 1418-1429.

[19] Zaman, Mohammad Faisal, et al. "A mode-matched silicon-yaw tuning-fork gyroscope with subdegree-per-hour Allan deviation bias instability." Journal of Microelectromechanical systems 17.6 (2008): 1526-1536.

[20] Zaman, Mohammad Faisal, Ajit Sharma, and Farrokh Ayazi. "The resonating star gyroscope: A novel multiple-shell silicon gyroscope with sub-5 deg/hr allan deviation bias instability." IEEE Sensors Journal 9.6 (2009): 616-624.

[21] Prikhodko, Igor P., et al. "Sub-degree-per-hour silicon MEMS rate sensor with 1 million Q-factor." 2011 16th International Solid-State Sensors, Actuators and Microsystems Conference. IEEE, 2011.

[22] Cho, J., J. A. Gregory, and K. Najafi. "Single-crystal-silicon vibratory cylinderical rate integrating gyroscope (CING)." 2011 16th International Solid-State Sensors, Actuators and Microsystems Conference. IEEE, 2011.

[23] Tao, Yi, et al. "Design, analysis and experiment of a novel ring vibratory gyroscope." Sensors and Actuators A: Physical168.2 (2011): 286-299.

[24] Cho, J., J. A. Gregory, and K. Najafi. "High-Q, 3kHz single-crystal-silicon cylindrical rate-integrating gyro (CING)." 2012 IEEE 25th International Conference on Micro Electro Mechanical Systems (MEMS). IEEE, 2012.

[25] Tatar, Erdinc, Said Emre Alper, and Tayfun Akin. "Quadrature-error compensation and corresponding effects on the performance of fully decoupled MEMS gyroscopes." Journal of Microelectromechanical Systems 21.3 (2012): 656-667.

[26] Prikhodko, I. P., A. A. Trusov, and A. M. Shkel. "North-finding with 0.004 radian precision using a silicon MEMS quadruple mass gyroscope with Q-factor of 1 million." 2012 IEEE 25th international conference on micro electro mechanical systems (MEMS). IEEE, 2012.

[27] Nitzan, S., et al. "Epitaxially-encapsulated polysilicon disk resonator gyroscope." 2013 IEEE 26th International Conference on Micro Electro Mechanical Systems (MEMS). IEEE, 2013.
[28] Prikhodko, Igor P., Alexander A. Trusov, and Andrei M. Shkel. "Compensation of drifts in high-Q MEMS gyroscopes using temperature self-sensing." Sensors and Actuators A: Physical201 (2013): 517-524.
[29] Ahn, C. H., et al. "Encapsulated high frequency (235 kHz), high-Q (100 k) disk resonator gyroscope with electrostatic parametric pump." Applied Physics Letters 105.24 (2014).
[30] Zotov, S. A., et al. "Self-calibrated MEMS gyroscope with AM/FM operational modes, dynamic range of 180 dB and in-run bias stability of 0.1 deg/hr." 2014 DGON Inertial Sensors and Systems (ISS). IEEE, 2014.
[31] Yang, Cheng, and Hongsheng Li. "Digital control system for the MEMS tuning fork gyroscope based on synchronous integral demodulator." IEEE Sensors Journal 15.10 (2015): 5755-5764.
[32] Zhao, Yang, et al. "A 0.57°/h bias instability 0.067°/√ h angle random walk MEMS gyroscope with CMOS readout circuit." 2015 IEEE Asian Solid-State Circuits Conference (A-SSCC). IEEE, 2015.
[33] Yoon, Sungjin, et al. "Tactical grade MEMS vibrating ring gyroscope with high shock reliability." Microelectronic Engineering 142 (2015): 22-29.
[34] Askari, S., et al. "Near-navigation grade quad mass gyroscope with Q-factor limited by thermo-elastic damping." Proc. Energy. Vol. 44. 2016.
[35] Askari, Sina, et al. "Vacuum sealed and getter activated MEMS Quad Mass Gyroscope demonstrating better than 1.2 million quality factor." 2016 IEEE International Symposium on Inertial Sensors and Systems. IEEE, 2016.
[36] Serrano, Diego E., et al. "Substrate-decoupled, bulk-acoustic wave gyroscopes: Design and evaluation of next-generation environmentally robust devices." Microsystems & Nanoengineering 2.1 (2016): 1-10.
[37] Wen, Haoran, Anosh Daruwalla, and Farrokh Ayazi. "Resonant pitch and roll silicon gyroscopes with sub-micron-gap slanted electrodes: Breaking the barrier toward high-performance monolithic inertial measurement units." Microsystems & nanoengineering 3.1 (2017): 1-9.
[38] Prikhodko, Igor P., et al. "Half-a-month stable 0.2 degree-per-hour mode-matched MEMS gyroscope." 2017 IEEE International Symposium on Inertial Sensors and Systems (INERTIAL). IEEE, 2017.
[39] Tatar, Erdinc, Tamal Mukherjee, and Gary K. Fedder. "Stress effects and compensation of bias drift in a MEMS vibratory-rate gyroscope." Journal of Microelectromechanical Systems26.3 (2017): 569-579.
[40] Li, Qingsong, et al. "0.04 degree-per-hour MEMS disk resonator gyroscope with high-quality factor (510 k) and long decaying time constant (74.9 s)." Microsystems & nanoengineering 4.1 (2018): 32.
[41] Giner, Joan, et al. "MEMS gyroscope with concentrated springs suspensions demonstrating single digit frequency split and temperature robustness." Journal of Microelectromechanical Systems 28.1 (2018): 25-35.
[42] Askari, Sina, et al. "Vacuum sealed and getter activated MEMS Quad Mass Gyroscope demonstrating better than 1.2 million quality factor." 2016 IEEE International Symposium on Inertial Sensors and Systems. IEEE, 2016.
[43] Cao, Huiliang, et al. "Design, fabrication and experiment of double U-beam MEMS vibration ring gyroscope." Micromachines 10.3 (2019): 186.
[44] Koenig, S., et al. "Towards a navigation grade Si-MEMS gyroscope." 2019 DGON Inertial Sensors and Systems (ISS). IEEE, 2019.
[45] Xu, Qiang, et al. "A tuning fork gyroscope with a polygon-shaped vibration beam." Micromachines 10.12 (2019): 813.
[46] Wen, Haoran, et al. "A Hermetically-Sealed 2.9 MHz $\mathrm {N}= 3$ Disk BAW Gyroscope with Sub-Degree-Per-Hour Bias Instability." 2020 IEEE 33rd International Conference on Micro Electro Mechanical Systems (MEMS). IEEE, 2020.
[47] Mojtaba, Hodjat-Shamami, and Ayazi Farrokh. "Eigenmode operation of piezoelectric resonant gyroscopes." Microsystems & Nanoengineering 6.1 (2020).
[48] Efimovskaya, Alexandra, Danmeng Wang, and Andrei M. Shkel. "Mechanical trimming with focused ion beam for permanent tuning of MEMS dual-mass gyroscope." Sensors and Actuators A: Physical 313 (2020): 112189.
[49] Cui, Jian, and Qiancheng Zhao. "A High-Performance Tactical-Grade Monolithic Horizontal Dual-Axis MEMS Gyroscope With Off-Plane Coupling Suppression Silicon Gratings." IEEE Transactions on Industrial Electronics 69.11 (2021): 11765-11773.
[50] Xu, Pengfei, et al. "A novel high-Q dual-mass MEMS tuning fork gyroscope based on 3D wafer-level packaging." Sensors21.19 (2021): 6428.
[51] Wang, Hao, et al. "A wafer-level vacuum packaged MEMS disk resonator gyroscope with 0.42°/h bias instability within±300°/s full scale." IEEE Transactions on Industrial Electronics 69.5 (2021): 5304-5313.
[52] Liu, Zhenming, Haoran Wen, and Farrokh Ayazi. "Multi-coefficient eigenmode operation—breaking through 10/h open-loop bias instability in wideband aluminum nitride piezoelectric BAW gyroscopes." Microsystems & Nanoengineering 9.1 (2023): 18.
[53] Parajuli, Madan, Guillermo Sobreviela, and Ashwin A. Seshia. "Silicon mems gyroscope with quatrefoil suspension system achieving 1 million quality factor." 2022 IEEE 35th International Conference on Micro Electro Mechanical Systems Conference (MEMS). IEEE, 2022.
[54] Serrano, Diego Emilio, et al. "0.25 deg/h closed-loop bulk acoustic wave gyroscope." 2022 IEEE International Symposium on Inertial Sensors and Systems (INERTIAL). IEEE, 2022.
